# Nutritional Disorders and Their Clinical Impact in Older Adults With COVID-19: A Retrospective Study From Qatar

**DOI:** 10.7759/cureus.107085

**Published:** 2026-04-15

**Authors:** AL Anoud Ali H Z AL Fehaidi, Shafi Hashmath Ulla Khan, Rana Abdeljubbar A Abdelrahman, Nisreen Mazin Saeed Skairjeh, Monica Doroja De Ramos, Pavithra Shine, Shakeel Ahmad Khan, Nesreen Talal Mohamed Hassan Ahel, Rudina Jehad Falah Alrefai, Nuseibah Mohammed Mourshid Alomari

**Affiliations:** 1 Dietetics and Nutrition/Geriatrics, Rumailah Hospital, Hamad Medical Corporation, Doha, QAT; 2 Geriatrics and Long-Term Care Department, Rumailah Hospital, Hamad Medical Corporation, Doha, QAT; 3 Dietetics and Nutrition/Geriatrics, Enaya Specialized Care Center, Hamad Medical Corporation, Doha, QAT

**Keywords:** aging, covid-19, nutritional disorders, outcomes, risk factors

## Abstract

Introduction

Older adults were at a higher risk of developing nutritional disorders, hospitalization, and death because of the coronavirus 2019 (COVID-19) pandemic. The burden of malnutrition and overnutrition in institutionalized older adults is a critical but under-researched factor of COVID-19 outcomes, particularly in the Middle East, where scarce data are available. The purpose of this study was to find out the prevalence of nutritional disorders among older patients with COVID-19 and to identify risk factors and clinical outcomes in Qatar.

Methods

A total of 55 adults aged over 60 years with confirmed COVID-19 diagnoses between March and August 2020 were identified from long-term care facilities and home-based health services in Qatar. Retrospective data on nutritional disorders, risk factors, and clinical outcomes were collected. SPSS Statistics version 28 (IBM Corp., Armonk, NY, USA) was used to analyze the data.

Results

The majority of the cohort were overnourished (n=34, 62%) or malnourished (n=16, 29%), and the rest of them had a normal nutritional status (n=5, 9%). The cohort was 60% females (n=33) and 40% males (n=22) and the mean age was 78.7+9.9 years. The malnourished cohort was associated with feeding problems (p<0.01), gastrointestinal symptoms (p<0.04), and nasogastric tube feeding dependency (p<0.003). Multivariate analysis showed the gastrointestinal symptoms as common predictors of nutritional disorders (p=0.03) and mortality (p=0.006). The mortality rate was 74% (n=41). The independent variables that were also associated with death were hydration status (p<0.001), infection (p<0.001), use of a nasogastric tube (p<0.002), and readmission (p<0.002).

Conclusions

The prevalence of nutritional disorders is high in older COVID-19 patients, and overnutrition (imbalanced nutrition) is more widespread than malnutrition. There is a high correlation between nutritional disorders and gastrointestinal symptoms, and mortality as well. Additional factors that were associated with mortality to a significant level were hydration status, infection, readmission, and the use of a nasogastric tube. Thus, it is essential to focus on the nutritional assessment and management of older COVID-19 patients and similar severe infections.

## Introduction

By 25 June 2023, the WHO Coronavirus dashboard indicated more than 500 million confirmed coronavirus 2019 (COVID-19) and 6.9 million deaths due to COVID-19 around the world [1]. This has had a special impact on the older adults who have been a substantial proportion of hospitalizations and had a higher mortality rate. Those aged 65 years and over were at a significantly higher risk with a response fatality rate near 30% worldwide [1]. This statistic translates to an estimated 1.85 million lives lost [1]. Comorbidities, decreased immune status, and concomitant geriatric syndromes have led to higher rates of hospitalization and death in the older adults [2]. Moreover, older adults with multiple medical comorbidities are particularly subject to exposure to an increased vulnerability to nutritional disorders.

Aging is associated with physiological changes and a change in the immune system. Calder et al. emphasize the importance of maintaining homeostasis of the immune system and balanced nutrition in defense against viral threats such as the one in the form of COVID-19 [3]. The pandemic has impacted food access and lifestyle, which has an adverse effect on the immune system and nutritional status. Older people are especially susceptible to contracting COVID-19 as they are weak, inactive, and under stress. Malnutrition is a recognized factor of adverse outcomes in older individuals with symptoms of COVID-19 and also has an important association with frailty and poor hospital outcomes in patients with acute diseases. Recinella et al. (2020) found that older COVID-19 patients with high nutritional risk as determined by the Geriatric Nutritional Risk Index (GNRI) have higher mortality [4]. Similarly, Pironi et al. (2021) reported that over 50% hospitalized patients with COVID-19, of whom 70% were aged above 65, were malnourished patients [5]. On the other hand, obesity and overnutrition are responsible for a long-lasting low-grade inflammatory condition that leads to a decreased immune system reaction towards COVID-19 [6]. Both undernutrition and obesity have been linked to higher levels of inflammation, and an undernourished and overweight person may be more prone to developing the severe form of COVID-19. Obesity also predisposes to comorbidities such as type 2 diabetes, hypertension, and cardiovascular disease, which also increase susceptibility and severity of COVID-19. Addressing the nutritional needs of older people during the COVID-19 pandemic for improved resilience and outcomes is a must.

Studies have shown malnutrition ranges from 27% to 52% [7-9], while overnutrition is 33-59% [10]. Malnutrition is frequently associated with factors such as advanced age, comorbidity, male gender, dependency, and social isolation [11]. In contrast, overnutrition has been linked to female gender, multimorbidity, psychological stress, medication use, and unhealthy lifestyle choices [12,13]. Both malnutrition and overnutrition adversely affect the overall health and well-being of older people, resulting in increased mortality rates, readmissions, morbidity, infections, and pressure injuries [14,15].

The magnitude of nutritional disorders among older adults has risen during the pandemic of the new coronavirus. Symptoms of infection with COVID-19 include vomiting, diarrhea, fever, abdominal pain, and shortness of breath, all of which can severely impact the nutritional status of the body [16,17]. Inadequate nutrition resulting from overnutrition or malnutrition may lead to higher susceptibility to infection and reduced recovery, which in turn complicates the management of people infected by COVID-19 in this vulnerable population.

This study sought to analyze the frequency of nutritional disorders among older people with COVID-19 and their associated factors. Additionally, the research examined the negative outcomes of these disorders within this population. The findings are supposed to guide the process of establishing effective interventions and healthcare practice improvements for older people with COVID-19.

## Materials and methods

Study design and population

Records of older patients diagnosed with COVID-19 infections between 1st March 2020 and 19th August 2020 were retrieved in retrospect. The study was conducted on people aged 60 years or over who were receiving care in long-term care facilities and/or home-based health services. Inclusion criteria included that patients were dietitian-assessed at the time of diagnosis of the COVID-19 infection. Patients with end-stage renal disease receiving hemodialysis, advanced hepatic disease, undergoing chemotherapy, or under palliative care were excluded from the study.

Patient demographics, co-morbidities, and blood parameters

Data was retrieved from electronic patient records from Rumailah Hospital, Doha, Qatar. Comprehensive demographic information (age, sex, functional status, comorbidities, prescribed medications, gastrointestinal symptoms, feeding problems and feeding routes) was collected. In addition, some relevant biochemical markers such as albumin, white blood cell count, and urea were retrieved in the electronic system and subjected to analysis.

Nutritional status

For all patients, measurements of height and weight were recorded. Body mass index (BMI) was derived based on a specific formula for each individual: weight (kg) divided by height (squared metres) [18].

Identification of malnutrition was done following the two-step approach set by Global Leadership Initiative on Malnutrition GLIM [19]. This method requires the presence of one phenotypic criterion, most commonly non-voluntary weight reduction or low BMI, in conjunction with one etiologic criterion, such as reduced food intake, reduced food assimilation, or a disease burden/inflammatory condition. The GLIM criteria divided malnutrition severity into two classes: moderate or severe.

The criteria established by the European Society of Parenteral and Enteral Nutrition (ESPEN) were utilized to diagnose malnutrition [20]. Undernutrition (malnutrition) was diagnosed based on ESPEN criteria. Both malnourished and non-malnourished people were assessed by dietitians with GNRI [21]. The GNRI scores were used as risk indicators for malnutrition and were classified as follows: No risk (score < 82), low risk (score 82-91), moderate risk (score 92-97), and severe risk (score >= 98).

The diagnosis of overnutrition or imbalanced nutrition was made among people with a BMI > 25 [18].

Nutritional outcomes

The study measured hydration status, skin condition (specifically, the presence of pressure injuries), and infection, such as pneumonia and urinary tract infections, within six months of the first admission with a confirmed case of COVID-19. Adverse health outcomes, morbidity (length of hospitalization, readmission rates), and mortality were documented within six months of index admission.

Statistical analysis

Data were evaluated based on descriptive statistics: Means and standard deviations for continuous variables, and percentages for categorical variables. Continuous variables were tested using Student's t-tests and categorical variables using the Pearson chi-square test. Multivariable general linear modeling was used to determine the predictors of nutritional disorders and mortality. Data analysis was performed with the statistical program SPSS Statistics version 28 (IBM Corp., Armonk, NY, USA) .

## Results

Table 1 summarizes the baseline characteristics of the study population. The participants were found to have a total of 55 older individuals who tested positive to COVID-19 at the time of the study. Among these participants, 60% were female (n=33) and 40% were male (n=22). The mean age was 78.7 ± 9.9 years. The average height and weight were 161 ± 7.8 cm and 68.7 ± 18.4 kg, respectively. Diabetes mellitus (n=43, 78%) and hypertension (n=46, 83%) were the most common comorbidities in the study population, after which there was dementia (n=21, 38%) and stroke (n=19, 34%). In 11% (n=6) cases the Charlson comorbidity score was 3 to 4 and in 89% (n=49) cases it was greater than 5. A large proportion of them (n=52, 94%) were on five or more drugs (polypharmacy) and bedridden (n=49, 89%). Over 50% of the patients (n=30, 54%) had pressure injuries. The prevalence of stage 2, 3 and 4 pressure injuries was 36% (n=11), 36% (n=11) and 26% (n=8) among the participants, respectively. The mean length of stay in the hospital was 15.98 ± 8.6 days and the mortality rate was 74% (n=41).

**Table 1:** Baseline characteristics (n=55) Results are expressed as mean±SD for continuous data and n, % & confidence interval for categorical data.

Characteristics	Number (n)	Percentage (%)	Confidence Interval
Age (years)	78.7±9.9	-	76-81
Height (Centimeters)	161±7.8	-	159-163
Weight (Kg)	68.7±18.4	-	63-73
Male	22	40%	27%-45%
Female	33	60%	54%-72%
Diabetes Mellitus	43	78%	64%-88%
Hypertension	46	83%	71%-92%
Coronary Heart Disease	13	23%	13%-37%
Dementia	21	38%	25%-52%
Stroke	19	34%	17%-59%
Charlson Comorbidity score (3-4)	6	11%	4%-22%
Charlson Comorbidity score (≥5)	49	89%	77%-95%
Polypharmacy (≥5 medications)	52	94%	84%-98%
Bedridden	49	89%	77%-95%
Feeding Dependence	28	51%	37%-67%
Hydration Status - Normal	10	18%	10%-30%
Hydration Status - Dehydration	25	45%	31%-59%
Hydration Status - Overhydration	20	36%	23%-50%
Pressure Injury	30	54%	40%-68%
Pressure injury stage 1	0	0	0
Pressure injury stage 2	11	36%	19%-56%
Pressure injury stage 3	11	36%	19%-56%
Pressure injury stage 4	8	26%	12%-45%
Length of hospital stay (days)	15.98±8.55	-	14-18.24
Mortality	41	74%	84%-98%

In Table 2, we describe the nutritional disorders and their associated factors in elderly people suffering from COVID-19. The evaluation showed that 62% of the cohort had overnutrition (n=34) and 29% malnutrition (n=16). Statistical comparisons did not demonstrate any significant differences between age, gender, anthropometric height, lean mass, percent weight change, diabetes, and dementia. Nevertheless, there was a significant difference in weight, with malnourished participants having significantly lower body weight than those in the normal and overnutrition categories (p=0.004). The BMI classification showed that all members of the malnutrition cohort were underweight, whereas all those classified as normal weight were within the normal BMI range. The overnutrition group included a significantly higher number of overweight or obese individuals (p<0.001). Feeding difficulties, including use of a denture, chewing difficulties, and dysphagia, were more common in the malnourished participants (p=0.01). Moreover, this group had an increased incidence of gastrointestinal manifestations, i.e., vomiting and diarrhea (p=0.04). Concerning the feeding modalities, nasogastric (NG) tube enteral nutrition was more common in the malnutrition group, while oral intake was more prevalent in both the normal and overnutrition categories (p=0.002 and p=0.003, respectively). No significant differences were found in serum albumin or urea values between the groups. Nutritional risk indices showed that most malnourished participants were at severe risk of malnutrition as per the GNRI and met the GLIM criteria for severe malnutrition. Hydration status was also different: the malnutrition cohort had a higher prevalence of dehydration, whereas the overnutrition group more often had normohydration (p=0.09). When exploring the adverse outcomes, the malnourished people had higher rates of pressure injuries, while the overnutrition had higher rates of readmissions (p=0.15 and p=0.05, respectively). Mortality did not differ significantly between the three groups (p=0.53).

**Table 2:** Characteristics amongst various nutritional disorders GNRI – Geriatric Nutritional Risk Index (Applicable only for undernutrition and normal persons) GLIM – Global Leadership Initiative on Malnutrition (Applicable only for undernutrition) PEG – Percutaneous Endoscopic Gastrostomy BMI – Body Mass Index GI symptoms – Gastro-Intestinal Symptoms SD – Standard Deviation

Characteristics	Malnutrition (n=16, 29%)	Normal (n=5, 9%)	Overnutrition or Imbalanced Nutrition (n=34, 62%)	P Value
Age (years), mean±SD	79.43±9.58	81.80±6.41	77.97±10.50	0.12
Male, n (%)	6 (38%)	2 (40%)	14 (41%)	0.97
Female, n (%)	10 (62%)	3 (60%)	20 (59%)	
Height (cm)	162±5.30	158±4.56	161±8.91	0.78
Weight (kg), mean±SD	58.35±15.21	57.40±5.07	74.93±18.34	0.004
Lean Mass, mean±SD	37.15±10.57	34.60±8.42	42.49±11.11	0.46
Percentage Weight Change last 6 months, mean±SD	12.34±5.04	14.73±6.91	11.91±4.50	0.27
BMI: Underweight (<18.5), n (%)	16 (100%)	0	0 (0%)	<0.001
BMI: Normal (23-25), n (%)	0 (0%)	5 (100%)	0 (0%)	
BMI: Overweight (25-29), n (%)	0 (0%)	0	14 (41%)	
BMI: Obesity (>30), n (%)	0 (0%)	0	20 (59%)	
Coronary Heart Disease, n (%)	4 (25%)	1(7%)	8 (23%)	0.97
Diabetes, n (%)	14 (87%)	4 (80%)	25 (73%)	0.53
Dementia, n (%)	3 (19%)	2 (40%)	16 (47%)	0.15
Charlson Comorbidity ≥5	15 (99%)	5 (100%)	29 (85%)	0.47
Polypharmacy (≥5)	14 (87%)	5 (100%)	33 (97%)	0.32
Feeding Problems: Denture, n (%)	9 (56%)	1 (20%)	6 (17%)	0.01
Feeding Problems: Chewing, n(%)	2 (12%)	4 (80%)	14 (41%)	
Feeding Problems: Swallowing, n (%)	5 (31%)	0	14 (41%)	
GI Symptoms: Vomiting, n (%)	4(25%)	2 (40%)	2 (6%)	0.04
GI Symptoms: Diarrhoea, n (%)	12(75%)	3 (60%)	32 (94%)	
Feeding Route: Oral, n (%)	6 (37%)	5 (100%)	28 (82%)	0.002
Feeding Route: Nasogastric Tube feeding, n (%)	9 (56%)	0	5 (15%)	0.003
Feeding Route: PEG feeding, n (%)	1 (6%)	0	1 (3%)	0.76
Albumin, mean±SD	26±5	29±8	24±6	0.36
Urea, mean±SD	39.98±94.90	12.98±8.31	11.46±8.65	0.17
GNRI- Low risk of Malnutrition	2 (12%)	0	n/a	0.45
GNRI- Moderate risk of Malnutrition	2 (12%)	1 (20%)	n/a	
GNRI- Severe risk of Malnutrition	12 (76%)	4 (80%)	n/a	
GLIM – Moderate Malnutrition	1 (6%)	n/a	n/a	0.33
GLIM – Severe Malnutrition	15 (94%)	n/a	n/a	
Hydration Status: Normal, n (%)	6 (37%)	0	4 (12%)	0.09
Hydration Status: Dehydration, n (%)	6 (37%)	4 (80%)	15 (44%)	
Hydration Status: Overhydration, n (%)	4 (26%)	1(20%)	15 (44%)	
Adverse outcomes: Pressure Injury, n (%)	6 (37%)	2(40%)	22 (64%)	0.15
Adverse outcomes: Infection, n (%)	8 (60%)	3(50%)	9 (26%)	0.14
Adverse outcomes: Length of stay (days), mean±SD	15.56±9.68	16.80±9.70	16.05±8.09	0.51
Adverse outcomes: Readmission, n (%)	8 (50%)	3 (60%)	28 (82%)	0.05
Adverse outcomes: Mortality, n (%)	11 (69%)	3 (60%)	27 (79%)	0.53

A multivariable general linear model was used to test the association between nutritional disorders and mortality as outlined in Table 3. The results show a statistically significant association between gastrointestinal symptoms (vomiting and diarrhea) and nutritional disorders (p=0.03). Likewise, these symptoms were significantly correlated with mortality (p=0.006). Lean mass was significantly associated with nutritional status (p=0.005) but was not related to mortality (p=0.53). Hydration status did not relate to nutritional disorders (p=0.45), but it was a significant predictor of death (p<0.001). The presence of an NG tube was not significantly associated with nutritional disorders (p=0.54), but it was significantly associated with mortality (p=0.002). Coronary heart disease showed no significant correlation with nutritional disorders (p=0.63) or death (p=0.10). The infection itself was not related to nutritional disorders (p=0.46), but it showed a strong predictive relationship with mortality (p<0.001). Pneumonia was significantly correlated with nutritional disorders (p=0.03) but was not significantly correlated with mortality (p=0.74). Finally, readmission was not associated with nutritional disorders (p=0.39) but was significantly associated with mortality (p=0.002).

**Table 3:** Variables associated with nutritional disorders and mortality in a multivariable general linear model.

Variables	Nutritional disorders P Value	Mortality P Value
Gastrointestinal symptoms (Vomiting and Diarrhea)	0.03	0.006
Lean Mass	0.005	0.53
Hydration Status	0.45	<0.001
Presence of Naso-Gastric Tube	0.54	0.002
Coronary Heart Disease	0.63	0.10
Infection	0.46	<0.001
Pneumonia	0.03	0.74
Readmission	0.39	0.002

## Discussion

This study showed the prevalence of nutritional disorders to be high in older adults with COVID-19. The prevalence of overnutrition was also found in a substantial percentage of people with overweight and obese status (n=34, 62%), which is consistent with the current evidence that shows that being overweight correlates with a higher risk of getting infected with coronavirus [22-24]. Malnutrition was found in 29% of the cohort (n=16). The risk of severe illness and mortality from COVID-19 is exacerbated by premature death risk factors such as non-communicable diseases (NCDs), including obesity, diabetes, and cardiovascular diseases [24,25]. These results are consistent with those in previous studies that report the prevalence of overnutrition [24] and malnutrition among the older population [9]. A small subset (n=5, 9%) did not have any nutritional disorders and may therefore have been relatively healthy nutritionally.

The cohort was older in average (mean age, 78.7 (±9.9) years) with high rates of comorbidities: mainly diabetes mellitus (n=43, 78%), hypertension (n=46, 83%), dementia (n=21, 38%) and stroke (n=19, 34%). These results agree with prior studies on the higher susceptibility of older people to infection with the coronavirus and their risk of developing complications as a result of their pre-existing comorbidities [26,27]. Previous research reports show that comorbidities are the leading cause in the background in 32-60%, with cardiovascular disease, hypertension, and diabetes being the most common [28,29]. The primary factors of vulnerability to COVID-19 are advanced age and underlying illnesses. Polypharmacy was observed in 94% (n=52) of the cases, and this is in line with the previous studies [30,31]. A total of 89% (n=49) of participants were bedridden. Previous studies have identified functional dependency as a recognized risk factor for coronavirus infection [32]. These findings are in line with research showing that frail and bedridden patients tend to suffer from poor nutrition, dementia, dehydration and other health complications even when suffering from mild infections. Without timely intervention, these risk factors could potentially contribute towards exacerbating health-related outcomes and an increase in mortality [33].

BMI was good at distinguishing between malnutrition, overnourished patients, and low BMI, in hospitalized older people with COVID-19 [4,9,24], all of them were associated with a higher risk of death [4,27]. The tube-fed patients were found to have an increased incidence of malnutrition, and this was in comparison with the oral-fed patients [34]. While the NG tube feeding was linked with overnutrition, tube-fed patients generally had undernutrition. Although previous studies indicate that malnutrition increases the risk of readmission [35], this study observed elevated readmission rates across all groups, with the highest rates among over-nourished individuals. Groups vulnerable to malnutrition were more likely to have feeding difficulties, use dentures, and have difficulty chewing and swallowing, suggesting barriers to adequate nutrition. The malnutrition group also experienced more gastrointestinal symptoms, such as vomiting and diarrhea, which further contribute to nutrient deficiencies. These results are supported by the literature, which shows weight loss, feeding difficulties, and gastrointestinal symptoms to be risk factors for malnutrition in older adults [7,11,36].

Apart from the high prevalence of nutritional disorders, during this study, a significant death rate was observed (n=41, 75%). A multivariable general linear model was used to identify factors that determine both nutritional disorders and death. Gastrointestinal symptoms such as vomiting and diarrhea were strongly related to nutritional disorders (p=0.03) and to mortality (p=0.006). Malnutrition in older people is likely aggravated by gastrointestinal symptoms related to the presence of COVID-19. The secretion of angiotensin-converting enzyme 2 (ACE2) is overexpressed in the digestive system and is a primary target of respiratory tract infection, as evidenced by the spread of COVID-19 [37]. Previous studies have shown that 16-50% of patients with confirmed COVID-19 infection experience one or more gastrointestinal symptoms during the course of the illness or at presentation [38]. A meta-analysis of 4,243 patients demonstrated a pooled prevalence of 17.6% of gastrointestinal symptoms but the quality of the population was poor because of the predominance of data from the Asian region [39]. Diarrhea and nausea/vomiting were experienced in less than 8% of patients in another meta-analysis, but these were not linked to increased mortality rates [40]. Another review and meta-analysis of 12,797 patients from 78 studies reported gastrointestinal symptoms in approximately 20% of patients, with diarrhea, loss of appetite, nausea and/or vomiting, and abdominal pain being the most frequent. Mortality rates for patients with gastrointestinal symptoms were comparable to general mortality rates [41]. Gastrointestinal symptoms can result in loss of nutrients and worsen nutritional deficiencies in older people. These symptoms can be further considered signs of general poor health and increased risk of adverse outcomes, including death. Even though previous reports have associated these symptoms with increased death rates in times of COVID-19, Wang et al. have refuted this correlation [42].

Individuals who had lower lean mass status were more likely to be malnourished or of normal weight rather than overweight or obese. Body composition is a critical factor in the development of malnutrition in older people, and hydration status is significantly associated with mortality risk, with a higher risk at lower hydration levels [43]. Dehydration is a serious health problem among older adults with various comorbidities, especially those who live alone. Poor hydration leads to susceptibility to infection and death [44] and often leads to long-term hospitalization and high mortality rates in this group of people [45].

The study had a small sample group as it was restricted to home healthcare or long-term care services. There were also some analytic constraints, such as small cell values and zero values, that limited the strength of the analysis. Moreover, the cross-sectional design does not allow the causal analysis of relationships between the identified factors and observed outcomes. The focus on older people with COVID-19 may also not allow the generalizability of findings to other populations or age groups.

## Conclusions

This study reports a high prevalence of nutritional disorders among older people with COVID-19 and identifies the main associated factors like gastrointestinal symptoms (vomiting and diarrhoea), lean mass, and pneumonia. Moreover, the mortality was associated with gastrointestinal symptoms, hydration status, presence of NG-tube, infection, and readmission to the hospitals. Thus, malnutrition and the overall health status of older people worsen and increase the risk of adverse outcomes.

Food and nutrition should be highlighted as a part of the public health measures, including sustainability efforts to enhance food systems and promote healthy eating. Nutritional evaluation and management for older adults with COVID-19 or other severe infections should employ holistic approaches that address patient needs and promote health and resilience throughout the course of illness and recovery.
